# Recurrent attack of acute myocardial infarction complicated with ventricular fibrillation due to coronary vasospasm within a myocardial bridge: a case report

**DOI:** 10.1186/s12872-020-01650-7

**Published:** 2020-08-24

**Authors:** Xingwei He, Zakarya Ahmed, Xin Liu, Chang Xu, Hesong Zeng

**Affiliations:** 1 Department of Cardiology, Tongji Hospital, Tongji Medical College, Huazhong University of Science and Technology, 1095# Jiefang Ave, Wuhan, 430030 China; 2 Department of Operation Room, Tongji Hospital, Tongji Medical College, Huazhong University of Science and Technology, Wuhan, China

**Keywords:** Vasospasm, Myocardial bridge, Myocardial infarction, Ventricular fibrillation, Implantable Cardioverter-defibrillator

## Abstract

**Background:**

Myocardial bridge (MB) often an inoffensive condition that goes in one or more of the coronary arteries through the heart muscle instead of lying on its surface. MBs sometimes leads to myocardial ischemic symptoms such as chest pain, even an occurrence of myocardial infarction. However, reports of severe and recurrent cardiac adverse events related to the MBs are rare.

**Case presentation:**

A 44-year-old male patient who suffered from a four-hour crushing chest pain ten years ago, was diagnosed as acute anterior ST-elevation myocardial infarction (STEMI). The initial findings of coronary angiography (CAG) showed MB was located in the middle part of the left anterior descending coronary artery (LAD). The patient was managed medically. Another re-attack of similar previous chest pain characteristics occured just after 3 days of discharge. Supra-arterial myotomy and CABG were the next adopted management. Postoperative progression was uneventful. However, 32 months after surgical treatment, the patient experienced an abrupt onset of chest pain accompanied by loss of consciousness. The ECG showed ventricular fibrillation (VF). After electrical cardioversion, an immediate CAG followed by CTA was performed which excluded thrombus or acute occlusion in the native coronary artery and an occlusion was observed at the end of the left internal mammary artery. An implantable cardioverter-defibrillator (ICD) was successfully performed for prevention of malignant arrhythmia. During ten years of follow-up, no complications have been identified.

**Conclusions:**

Although MB is mostly benign, it may lead to significant cardiovascular consequences. Supra-arterial myotomy is an appropriate treatment option for this patient who failed to optimal medical therapy. Furthermore, ICD implantation must be considered in order to prevent malignant ventricular arrhythmia caused by continuous spasm resulting in ischemia. Further investigations are required to confirm the clinical effectiveness of these procedures.

## Background

The coronary arteries are commonly lying on the surface of the myocardium. A segment of one or more of the major coronary arteries that have an anomaly characterization of a typical intramyocardial direction is defined as a myocardial bridge (MB, usually located in the middle segment of the LAD). MB may lead to a systolic luminal narrowing, which is often delayed to the diastolic period, especially during heavy exercise that causes a reduction of coronary artery blood perfusion [1]. Due to the specific hemodynamic states caused by MB, the rate of proximal coronary atherosclerosis is 86%. MB was first reported by Reyman in 1737, and then it was confirmed angiographically by Porstmann and Iwig in 1960 [2]. The prevalence has been reported to range from 5.4–85% in autopsy series to 0.5–29.4% on coronary angiography [3].

On CAG, MB typically presents as a systolic narrowing, or “milking” of the artery, with a “step-down” and “step-up” demarcating the influenced area, and partial or complete decompression in the diastolic period [4]. Although MB can be found in any epicardial coronary artery, it commonly involves the mid-portion of the LAD [5]. MB is often considered as a benign variation due to the fact that the narrowing effect of the vessel commonly occurs during systole, while most of the myocardial demand for blood flow occurs during the diastole. Rarely,the compression of a coronary artery by the MB can be associated with clinical events of myocardial ischemia and even cause myocardial infarction, malignant arrhythmias, or sudden cardiac death (SCD).

In this article, we describe a patient who presented with a history of recurrent episodes of myocardial infarction, resulting from vasospasm within MB located in the mid-segment of the LAD, which then complicated to ventricular fibrillation. We report the results of the therapeutic strategy and long-term outcome.

## Case presentation

**Fig. 1:**
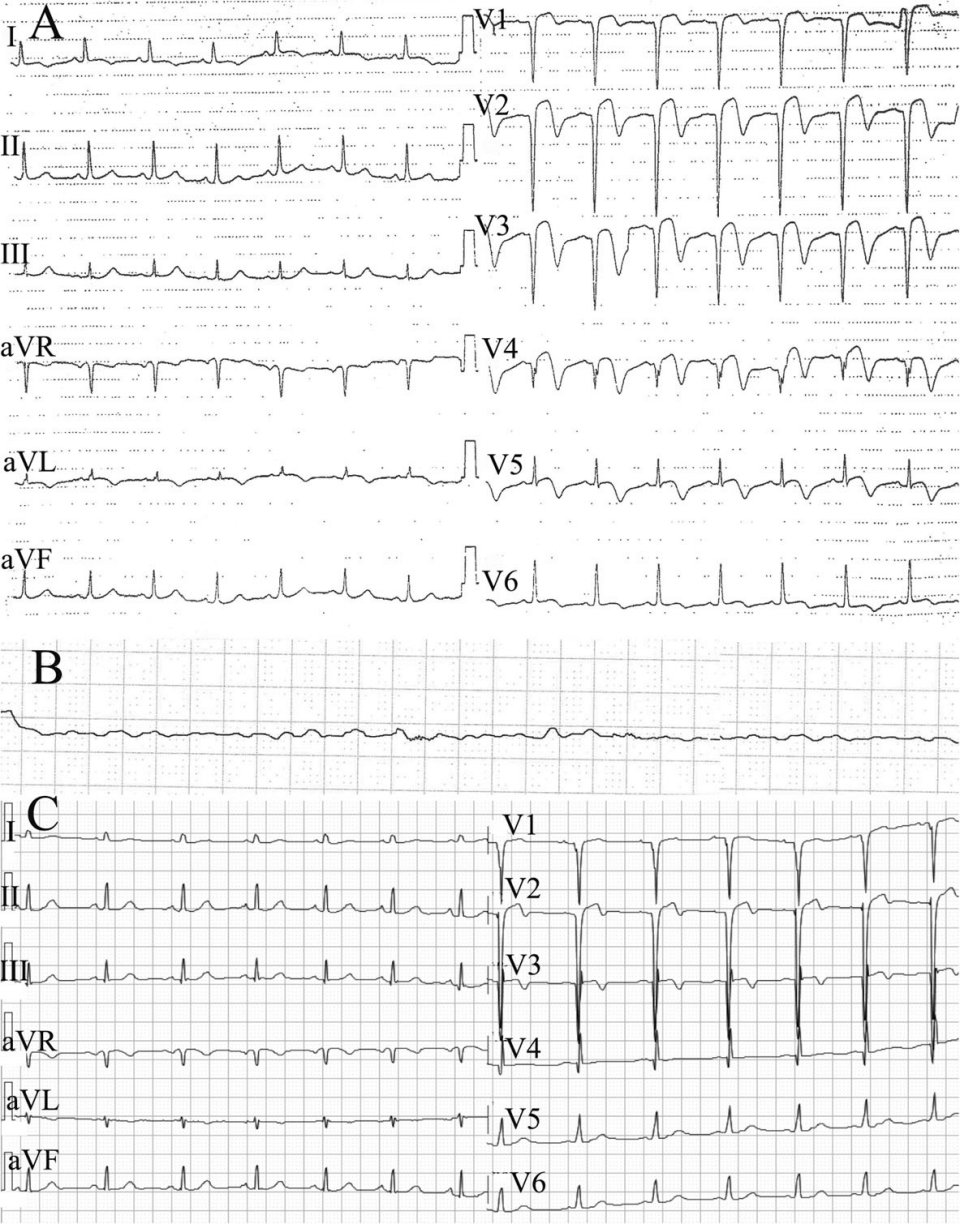
The first ECG revealed typically convex ST-segment in the V1 to V4 leads, followed by inverted T waves in the precordial leads (**a**); the second ECG recorded from a cardiac monitoring 32 months after Supra-arterial myotomy and CABG management showed VF (**b**); ECG was obtained after ICD implantation during patient follow-up (**c**)

**Fig. 2:**
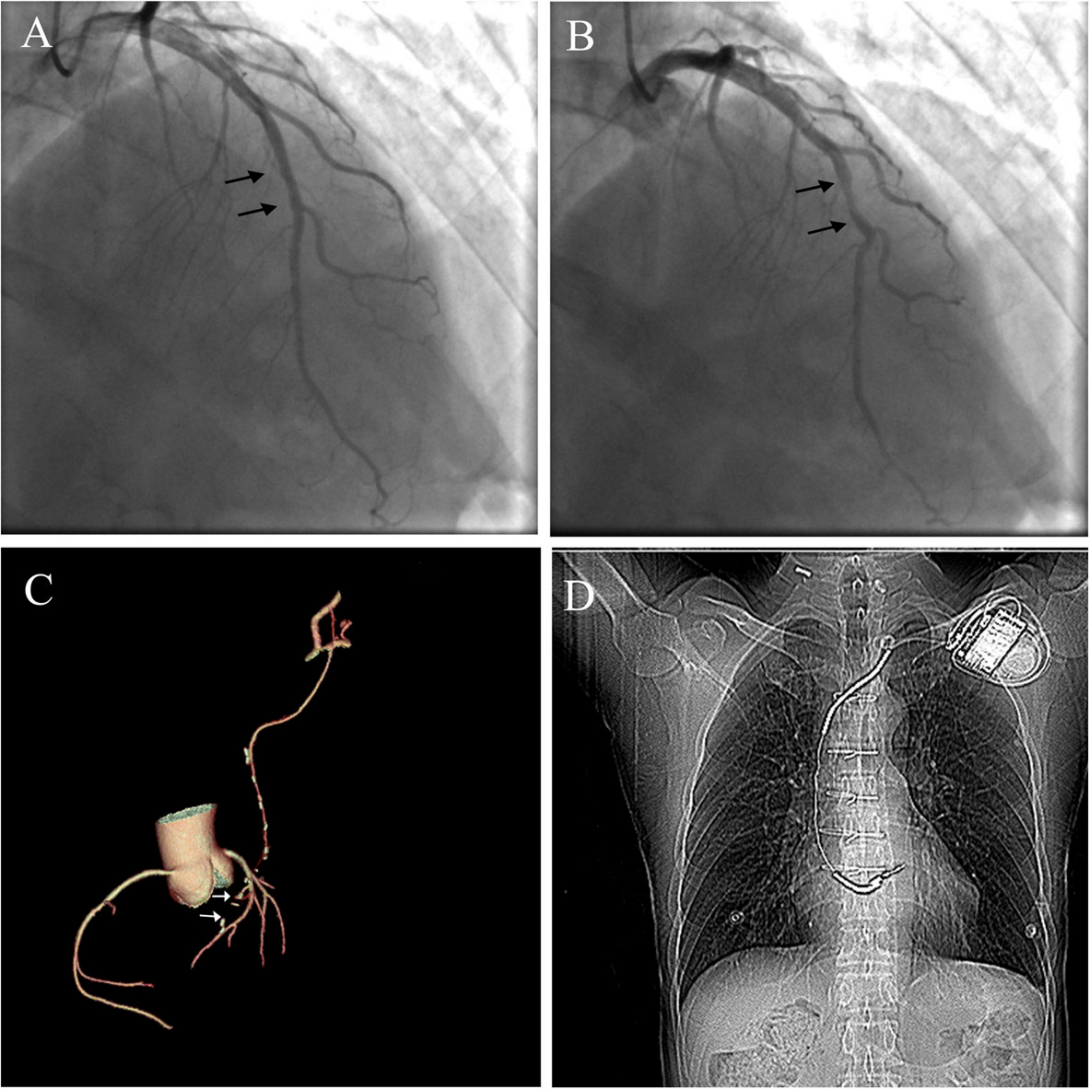
RAO cranial view of the LAD at baseline displaying MB segment during systolic period (**a** black arrows), and diastolic period (**b** black arrows); volume-rendered 3-dimensional reconstruction displaying occlusion at the end of the LIMA (**c** white arrows); a chest radiograph showing the ICD device and its lead location **d**

A 44-year-old male patient presented to the emergency department of our hospital ten years ago with crushing chest pain persisting for 4 h after heavy labor. Hehad 5 years history of hypertension and no history of diabetes, smoking, and substance abuse. Physical examination revealed normal blood pressure in arms and legs with no evidence of an arm-leg gradient. Initial electrocardiography (ECG) showed elevated ST-segment (1-4 mm) at the anterior leads V_1_ to V_6_ (Fig. 1a). Blood test showed elevated myocardial troponin I levels of 0.5 ng/ml (normal range 0.00–0.04 ng/ml), creatine kinase-MB of 11.9 ng/ml (normal range 0.3–4.3 ng/ml). The patient was diagnosed as acute anterior ST-elevation myocardial infarction and then underwent emergency catheterization. The initial findings of the CAG showed approximately 10 mm in length, with 50% vascular stenosis during the systolic period in the middle part of theLAD and normal during the diastolic period (Fig. 2a-b). The antegrade blood flow in the LAD distal to the MB segment was normal (TIMI III) without any evidence of thrombus or spontaneous coronary artery dissections. All the other coronary vessels appeared normal. The patient’s symptoms were stable, and was discharged after 1 week of hospitalisation. However, merely three days post-discharge, the patient suffered from another episode of chest pain despite the use of β-blocker (metoprolol 47.5 mg once a day) and calcium channel blockers (dihiazem 90 mg twice a day). He had an elevated myocardial troponin I level of 30 ng/ml. ECG showed elevated ST-segment at the precordial leads V_1_-V_6_, echocardiogram reported abnormal segmental wall motion with an ejection fraction of 45%. The patient was diagnosed as a recurrent of STEMI.Supra-arterial myotomy, and CABG management were the treatment choice, which was successfully performed through a median sternotomy. Post-discharge medications was prescribed taking cardiovascular risk factors into consideration. Thereafter, no special discomfort was reported.

Thirty-two months after the surgery, the patient experienced another attack of chest pain in the morning along with a loss of consciousness on his way to the hospital in an ambulance. Cardiac monitoring showed VF (Fig. 1b). Cardiopulmonary resuscitation followed by electrical cardioversion restored normal sinus rhythm. He had two further episodes of VF at the hospital which was electrically cardioverted to a borderline ventricular autonomous rhythm and eventually sinus rhythm. A prompt CAG followed by coronary CTA was performed which confirmed an occlusion at the end of the LIMA (Fig. 2c). The time of occlusion was not clear. Based on these findings, we considered ischemia-induced arrhythmia precipitated by coronary vasospasm as the most likely cause. According to the most current recommendation guidelines [6], an ICD (Fig. 2d) was implanted to prevent SCD as a consequence of malignant ventricular arrhythmia. The patient was discharged on aspirin, statin, beta-blockers, and calcium channel blocker.

During ten years of follow-up, the patient has never experienced any angina or myocardial infarction. The ICD readings demonstrated no ventricular arrhythmias (Fig. 1c). He is still under close and long-term follow-up.

## Discussions and conclusions

MB is a common anatomical anomalyand mostly involves mid to distal parts of the LAD. On IVUS, the tunneled segment of LAD is of variable degree of compression that persists into diastole with the typical finding of the “half-moon phenomenon”. For a long time, MB has been considered as a benign anatomic variation since the pressing of the coronary artery independently occurs during systole, which contains minimal blood perfusion to the heart muscle. However, several studies have supported the fact that MB can induce some fatal conditions, such as coronary thrombosis, spasm, acute coronary syndrome, transient ventricular dysfunction, takotsubo syndrome, life-threatening ventricular arrhythmias and/or SCD [7, 8].

There are several proposed mechanisms over which MB is supposed to cause myocardial ischemia and related symptoms. First, phasic systolic compression of the coronary artery leads to a persistent mid-to-late diastolic reduction in the vessel diameter. During exertional tachycardia, the relatively greater shortening of diastole than systole and the reduction of ventricular filling causes a decrease in coronary flow leading to myocardial ischemia [9–11]. Secondly,“milking” may lead to systolic retrograde intracoronary flow and complete blood flow disruption in the coronary artery with increased shear stress. This is a cause for the characteristic progression of atherosclerosis in the proximal segment of the MB, whereas the bridged segment stays relatively free from atherosclerosis [12]. Finally, the dysfunction of coronary endothelial and coronary vasospasm has also been proposed as an important mechanism for myocardial ischemia [13].

The current case report discusses a recurrent myocardial infarction with complicated ventricular fibrillationdue to coronary spasm in the MB site. CAG did not reveal fixed arteriosclerotic lesion in LAD but found that there was MB, which displayed stenosis during the systole period. Although a provocation test was not attempted during CAG, considering the patient’s symptoms and ECG findings, webelieve the MB was responsible for the coronary vasospasm.

Currently, the management of MB in symptomatic patients is still argumentative. First-line treatment of the symptoms secondary to MB consists of calcium-channel blockers and beta-blockers [14]. Nitratesare contraindicated because of the excessive contraction due to secondary tachycardia and reflex sympathetic nerve activation [15]. For the intractable symptoms that responded poorly to conservative medical treatment, other therapeutic options have been attempted, such as coronary stent, supra-arterial myotomy, and CABG. However, despite the fact that coronary stent implantation may improve the abnormal hemodynamics and recover symptoms no research had verified the perfusion stability of the myocardium when a perfusion defect was present before stent implantation.

Surgical management should be the first choice for patients who have symptoms related to MB and have failed optimal medical therapy [16]. The options for the surgical management of MB include supra-arterial myotomy, CABG, or both [17, 18]. Surgical technique choices depend on MB features and local hospital competency. Supra-arterial myotomy management help to reduce patient symptoms, prevents the incidence of local myocardial ischemia and increases coronary blood flow, but there are risks such as ventricular aneurysm formation, wall perforation, and post-operative subendocardial hemorrage. Long-term complications such as myotomy scarring, specifically in MBs that lay deeper, may occur on the area over the entire artery resulting in recurrence of compression. These risks and complications could be avoided by bypassing the MB segment, which has the advantage of alleviating future ischemia by undergoing a CABG surgery [19]. However, long-term bypass patency secondary to competitive flow may be affected when bypass and myotomy are performed without proximal coronary occlusion. Important points to be noted are that: supra-arterial myotomy is the first choice for symptomatic patients who have failed to achieve optimal medical therapy. Bypass surgery should be performed in combination with or instead of supra-arterial myotomy when there is a proximal coronary obstruction or if there are technical considerations that preclude myotomy (e.g., extensive, deep, or tortuous MB). Due to the relatively deep location of the MB in the current case, bypass surgery combined with myotomy was carried out to achieve collected benefits of both surgical techniques and prevent further complications. However, the distal CABG graft was occluded, probably due to competitive flow. For patients who have experienced VF due to vasospasm, an ICD implantation might be a therapeutic option for preventing SCD.

The current report highlights the association between MB and significant cardiovascular consequences. Supra-arterial myotomy is an appropriate treatment option for symptomatic patients who have failed optimal medical therapy. For patients who experienced a ventricular fibrillation arrhythmic complication, an ICD implantation is the suitable therapeutic option for the secondary prevention of SCD. However, the best treatment strategy for patients with MB still requires more clinical case evaluations and further randomized study.

## Data Availability

All relevant data supporting the conclusions of this article are included in the article.

## Abbreviations

MB: Myocardial bridge
CABG: Coronary artery bypass grafting
CAG: Coronary angiography
LAD: Left anterior descending coronary artery
LIMA: Left internal mammary artery
ICD: Implantable cardioverter-defibrillator
CTA: Computed tomography angiography
VF: Ventricular fibrillation
SCD: Sudden cardiac death
ECG: Electrocardiography
TIMI: Thrombolysis in myocardial infarction
IVUS: Intravascular ultrasound
